# Retrospective study on canine idiopathic epilepsy treatment in primary care practices in the United States

**DOI:** 10.3389/fvets.2026.1723038

**Published:** 2026-02-09

**Authors:** Elisa Pompermaier, Jo Ann Morrison, Fabio Stabile, Luisa De Risio

**Affiliations:** 1 Wear Referrals Small Animal Hospital, Part of Linnaeus Veterinary Limited, Mars Veterinary Health, Bradbury, United Kingdom; 2 Mars Veterinary Health, Vancouver, WA, United States; 3 Mars Veterinary Health, Friars Gate, Solihull, United Kingdom

**Keywords:** anti-seizure drugs, canine idiopathic epilepsy, first-line therapy, seizure management, therapeutic drug monitoring

## Abstract

The aim of this study is twofold: to characterize the epidemiology of idiopathic epilepsy (IE) in a large US primary care provider, and to investigate primary care veterinarians’ anti-seizure drugs (ASDs) prescribing practices. A multicenter retrospective study was conducted: Banfield Pet Hospital electronic medical records were searched (01/01/2020–31/12/2023) for dogs aged 6 months to 6 years at first recorded epileptic seizure, with normal general and neurological examinations and unremarkable blood analyses. To further support the IE diagnosis, only dogs prescribed ASDs were included. Signalment, ASDs name and dosing were recorded. Eight-hundred-fifty-three dogs met the inclusion criteria, corresponding to a prevalence of 0.03% (853/2,969,209 over 4 years). Labrador Retriever, Chihuahua and Siberian Husky were the most represented breeds. The median age at diagnosis was 3.3 years. Males accounted for 60.6% of cases. Phenobarbital (34.9%) and levetiracetam (31.3%) were the most prescribed first-line-ASDs, followed by zonisamide (22.9%) and potassium bromide (11.1%). Phenobarbital median maintenance dose was 2.5 mg/kg (IQR 2.2–3.0 mg/kg) per os (PO), with 99.3% of cases receiving it every 12 h. Extended-release levetiracetam was used in 97.4% of cases; the median dose was 29.7 mg/kg (IQR 24.1–34.6 mg/kg) PO, prescribed every 12 h in 87.7% of dogs. Zonisamide was prescribed at a median dose of 5.8 mg/kg (IQR 4.7–7.4 mg/kg) PO every 12 h (98.0%). Potassium bromide maintenance median daily dose was 29.9 mg/kg (IQR 24.0–38.4 mg/kg) PO with a once-daily administration in 83.3% of dogs. Phenobarbital serum concentration was monitored in 77.5% of cases, while bromide serum concentration was monitored in 31.6% of dogs. The estimated prevalence was lower than previous studies, possibly due to strict diagnostic inclusion criteria and data extraction limitations. Labrador Retrievers were the most affected breed, while other predisposed breeds were underrepresented among IE cases. Prescribing practices generally aligned with the ACVIM guidelines, suggesting good implementation of this knowledge in one of the largest US primary care providers. The variability in levetiracetam and potassium bromide dosing highlights the need for updated evidence-based dosing guidelines, while education on the value of therapeutic drug monitoring could support veterinary and pet-owner’s decision making in the management of IE.

## Introduction

Canine idiopathic epilepsy (IE) is the most prevalent chronic neurological disorder in dogs, affecting an estimated 0.6% of the general canine population in the United Kingdom (1). However, breed-specific prevalence can be considerably higher, reaching up to 20% in Belgian Shepherds in the United States (US) (2, 3) and as high as 33% in certain breeding lines in Denmark (4).

Canine IE is a brain disorder for which the nature of the underlying cause remains unknown (5). A genetic cause is often suspected based on family history (i.e., family members with IE) and a prevalence in the breed higher than in the general canine population (2–7).

In dogs with IE, the age of onset of epileptic seizures is between 6 months and 6 years, the interictal physical and neurological examination are unremarkable, and blood tests reveal no epileptogenic abnormalities (8). Lack of abnormalities on magnetic resonance imaging (MRI) of the brain and cerebrospinal fluid (CSF) analysis enable a higher confidence level in the diagnosis of IE (8).

In the primary care setting, access to advanced imaging modalities and CSF analysis is generally limited. As a result, IE is typically presumed based on the age of onset of recurrent epileptic seizures, medical history, clinical presentation and blood tests to rule out reactive seizures and other causes of epileptic seizures. Notably, recent evidence has questioned the diagnostic yield of brain MRI and CSF analysis in such cases. In a study including more than 400 dogs with recurrent epileptic seizures, unremarkable neurological examination and normal routine blood analysis, the brain MRI revealed no structural abnormalities in dogs under 1 year of age, while detectable lesions were identified in only 1.6% of dogs aged between one and 6 years (9). Such findings suggest that structural epilepsy is uncommon in this population, supporting IE as the predominant etiology. Similarly, another study in dogs with suspected IE (defined by recurrent epileptic seizures, normal interictal physical and neurological exam, unremarkable blood tests and brain MRI), found that the likelihood of detecting an underlying cause for the epileptic seizures was not increased when performing CSF analysis (10).

Management of idiopathic epilepsy (IE) is primarily based on the use of anti-seizure drugs (ASDs) (11, 12). The American College of Veterinary Internal Medicine (ACVIM) Consensus Statement on Seizure Management in Dogs (12), together with a systematic review by Charalambous et al. (13), critically evaluated the available evidence on anti-seizure drug (ASD) efficacy in order to provide evidence-based recommendations for their use. Based on the evidence available at the time, the 2015 ACVIM Consensus Statement (12) concluded that phenobarbital (PB), imepitoin, and potassium bromide (KBr) had the strongest evidence supporting their efficacy in the management of IE in dogs, whereas the evidence for levetiracetam (LEV) and zonisamide (ZNM) was weaker. The same Consensus Statement also highlighted the value of therapeutic drug monitoring for PB, KBr and ZNM to optimize efficacy and minimize occurrence of toxicity (12).

Regulatory frameworks further influence anti-seizure drug (ASD) prescribing practices, with notable differences between the US and Europe. In the US, only KBr and PB hold conditional Food and Drug Administration approval for the treatment of IE in dogs, while imepitoin has been licensed since 2018 exclusively for management of noise aversion (14–16). Nevertheless, imepitoin (when used for the management of epilepsy) along with LEV and ZNM, may still be prescribed under the Animal Medicinal Drug Use Clarification Act, which allows extra-label use of approved human and veterinary medicines within a valid veterinary–client–patient relationship (17, 18). In contrast, within the United Kingdom and European Union, PB (19), KBr (20) and imepitoin (21) are all licensed specifically for the treatment of epilepsy in dogs, while LEV and ZNM are only available as human drugs and may be prescribed to dogs under the veterinary “Cascade” system (22, 23).

It is currently unknown how prescribing practices for the treatment of presumed IE in the US conform to the ACVIM Small Animal Consensus Statement on Seizure Management in Dogs (12).

The primary aim of this study was to investigate primary care veterinarians’ ASDs prescribing preferences by collecting observational data on the most frequently used therapy in a large primary care provider in the US, while also describing the epidemiology of IE within the study population.

## Materials and methods

A multicenter retrospective study was performed using electronic medical records from Banfield primary care practices throughout the US. The study period extended from 1 January 2020 through 31 December 2023.

Medical records were obtained from the Banfield primary veterinary care network, comprising more than 1,000 primary care small animal hospitals across 42 US States, the District of Columbia, and Puerto Rico.

For the purpose of this study, IE was defined as epileptic seizure onset between 6 months and 6 years of age in dogs with normal clinical and neurological examinations, unremarkable routine blood analyses and documentation of an ASD prescription. The Banfield electronic medical record system has harmonized coding for clinical presentations and diagnosis; dogs with seizures were identified using specific coding (“Seizures,” “Seizures, Acquired,” “Seizure, Active” and “Seizures, Idiopathic”). Based on this definition and the standardized coding system, the Banfield database was searched to identify canine patients aged 6 months to 6 years of age at the time of the first recorded epileptic seizure, with a normal general and neurological examination, and blood analyses (hematocrit, total protein, blood urea nitrogen, alkaline phosphatase, alanine transaminase, albumin, creatinine and blood glucose) revealing no epileptogenic abnormalities (5, 8, 24). To further support the IE diagnosis, only dogs that were recorded as having prescribed ASDs were included and medical record data was reviewed for 365 days after initial diagnosis. To estimate population prevalence, the denominator consisted of all unique canine patients aged 6 months to 6 years presented to Banfield practices during the study period.

The following data were extracted from electronic medical records of dogs visiting Banfield: breed, body weight, age, sex, neutering status, body condition score (BCS), and blood test results. Laboratory data were reviewed including hematocrit, total protein, blood urea nitrogen, alkaline phosphatase, alanine transaminase, albumin, creatinine, blood glucose, PB serum concentration and bromide serum concentration.

Records of the prescribed ASDs were reviewed for the 365-day period following the initial presentation for epileptic seizures. Data collection was limited to the first-line oral ASD prescribed. In cases where more than one first-line ASD was prescribed (at treatment initiation), the dosage of each medication was included in the analysis. Oral ASDs included in the study were PB, LEV, KBr and ZNM. For each prescription, the following details were recorded: drug name, formulation and frequency of administration.

Data on breed, age, sex, weight and body condition score were summarized as percentages (for categorical data) or by median, range and interquartile range (IQR; for continuous data).

The percentage of cases with recorded PB and bromide serum concentration was calculated. For each ASD, the administered dose (mg/kg) was manually calculated for every dog. Dosage calculations were derived by dividing the total milligram amount prescribed by the recorded body weight (kg) at the time of prescription. Doses and frequencies of PB, LEV (extended and fast-release), and ZNM were summarized as for continuous data above. Cases of KBr treatment were divided into those involving maintenance or loading doses, with doses and frequencies in each category summarized.

To evaluate whether the prescribed ASD regimens were aligned with ACVIM Consensus Statement recommendations (12), the dose, dose range and dosing interval for each ASD were compared.

Only numeric and coded data from the Banfield electronic medical records were available for analysis; unstructured free-text notes were inaccessible due to privacy policies and limitations on data extraction and analysis. As a result, information on seizure dates and treatment response could not be evaluated.

## Results

During the four-year study period, 853 of 2,969,209 dogs (aged between 6 month and 6 years of age) seen at Banfield primary care practices met the inclusion criteria. The prevalence of IE was estimated at 0.03% (853/2,969,209).

The median age at the time of IE diagnosis was 3.3 years (range 6 months-6 years; IQR 2.1–4.6 years). Males accounted for 60.6% (517/853) of cases, of which 78.5% (406/517) were neutered. The proportion of females in the cohort was 39.4% (336/853), with 86.6% (291/336) being sterilized.

The median body weight was 18.7 kg (range 0.5–66.5 kg; IQR 8.4–28.8 kg). Body condition score was reported on a 9-point scale (25, 26), with 62.8% (535/852) of dogs scoring 5/9 at presentation (SD: 0.9).

Labrador Retriever (8.3%), Siberian Husky (7.5%), Chihuahua (6.8%), Crossbreed (6.6%), Poodle (5.0%), German Shepherd (4.7%) and Australian Shepherd (4.3%) were the most prevalent breeds amongst the IE population (Table 1).

**Table 1:** Demographic information.

Variable	Results
Breeds (top 15)	Labrador Retriever: 8.3% (71/853)
	Siberian Husky: 7.5% (64/853)
	Chihuahua: 6.8% (58/853)
	Crossbreed 6.6% (56/853)
	Poodle: 5.0% (43/853)
	German Shepherd: 4.7% (40/853)
	Australian Shepherd: 4.3% (37/853)
	Pit Bull: 3.9% (33/853)
	Beagle: 3.4% (29/853)
	Shih Tzu: 3.1% (26/853)
	Golden Retriever: 2.9% (25/853)
	Yorkshire Terrier: 2.9% (25/853)
	French Bulldog: 2.7% (23/853)
	Dachshund: 2.7% (23/853)
	Maltese: 2.3% (20/853)

First-line treatment ASDs were PB (298/853 dogs, 34.9%), LEV (267/853 dogs, 31.3%), ZNS (195/853 dogs, 22.9%), and KBr (95/853 dogs, 11.1%) (Tables 2, 3). In two cases, a combination of ASDs was dispensed as first-line treatment: one dog was prescribed extended-release (XR) LEV (52 mg/kg every 12 h) and PB (2 mg/kg every 12 h), while the other case received KBr (17 mg/kg every 24 h) and PB (2 mg/kg every 12 h). The patient receiving LEV and PB was issued a 30-day prescription for each drug. In contrast, the dog on KBr and PB was provided a 30-day course of PB, while the duration of KBr treatment was unspecified.

**Table 2:** Summary of phenobarbital (PB), levetiracetam (LEV) and zonisamide (ZNS) doses (mg/kg) and frequency of administration in 752 dogs (MD: maintenance dose: LD: loading dose; XR: extended release; IQR: interquartile range).

Drug	Number of dogs	Median dose (mg/kg)	IQR (mg/kg)	Minimum (mg/kg)	Maximum (mg/kg)	Percentage of dogs receiving it every 24 h	Percentage of dogs receiving it every 12 h	Percentage of dogs receiving it every 8 h	Percentage of dogs receiving it every 6 h
Phenobarbital MD	294	2.5	2.2–3.0	0.6	5.8	0.7% (2/294)	99.3% (292/294)	0%	0%
Phenobarbital LD	1	4.1	n/a	4.1	4.1	0%	0%	0%	100% (1/1)
Levetiracetam XR	260	29.7	24.1–34.6	11.0	151.0	2.3% (6/260)	87.7% (228/260)	10.0% (6/260)	0%
Levetiracetam fast-release	2	36.8	n/a	22.7	104.0	0%	50% (1/2)	50% (1/2)	0%
Zonisamide	195	5.8	4.7–7.4	1.0	16.1	2.0% (4/195)	98.0% (191/195)	0%	0%

Dose (mg/kg) data were available for 98.7% (294/298) of dogs on PB, 98.9% (264/267) of dogs on LEV and 100% (195/195) of dogs on ZNS. Frequency of administration data were available for 99.0% (295/298) of dogs on PB, 98.1% (262/267) of dogs on LEV and 100% (195/195) of dogs on ZNS. Only 2 of the dogs were concurrently administer two anti-seizure drugs (PB and LEV, PB and potassium bromide).

**Table 3:** Summary of potassium bromide daily dose (mg/kg) in 92 dogs receiving potassium bromide (MD: maintenance dose; LD: loading dose; IQR: interquartile range).

Drug	MD or LD	Number of dogs	Median dose (mg/kg)	IQR (mg/kg)	Minimum (mg/kg)	Maximum (mg/kg)
Potassium bromide daily dose	MD	66	29.9	24.0–38.4	11.5	52.0
	LD	26	93.7	64.6–126.7	51.3	551.2

Dose (mg/kg) and frequency of administration data were available for 96.8% (92/95) of dogs on potassium bromide.

Phenobarbital was typically prescribed at an initial median maintenance dose of 2.5 mg/kg (range 0.6–5.8 mg/kg; IQR 2.2–3.0 mg/kg), prescribed every 12 h in 99.3% of cases. The remaining 0.7% of cases received PB every 24 h, at a dose of 4.2 mg/kg and 5.2 mg/kg, respectively. One patient was prescribed a PB loading protocol (LD) of 4.1 mg/kg every 6 h for 24 h for a total of 24 mg/kg in 24 h (Table 2). Phenobarbital serum concentration monitoring was performed in 77.5% of cases.

Levetiracetam was the second most frequently prescribed ASD, with the XR formulation used in 97.4% of dogs. Extended-release levetiracetam presented a wide dose range, with a median of 29.7 mg/kg (range 11.0–151.0 mg/kg; IQR 24.1–34.6 mg/kg). It was prescribed every 12 h in 87.7% of dogs (median dose 29.8 mg/kg every 12 h; range 11.0–79.3 mg/kg; IQR 24.2–34.2 mg/kg), every 8 h in 10.0% of cases (median 25.8 mg/kg every 8 h; range 16.9–151.0 mg/kg; IQR 20.8–31.6 mg/kg) and every 24 h in the remaining 2.3% (median dose 39.9 mg/kg every 24 h; range 16.6–48.4 mg/kg; IQR not calculated due to small sample size). The fast-release formulation was used in the remaining 2.7% of dogs receiving LEV, at a median dose of 36.8 mg/kg (range 22.7–104.0 mg/kg; IQR not calculated due to small sample size), prescribed every 12 or 8 h in equal proportions (Table 2).

Zonisamide was prescribed at a median dose of 5.8 mg/kg (range 1.0–16.0 mg/kg; IQR 4.7–7.4 mg/kg), with 98.0% of dogs receiving it every 12 h and 2.0% receiving it every 24 h (Table 2).

Potassium bromide was prescribed at a median maintenance dose of 29.9 mg/kg/day (range 11.5–52.0 mg/kg/day; IQR 24.0–38.4 mg/kg/day) in 71.7% (66/92) of cases. Once-daily dosing was used in 83.3% (55/66) of dogs, with the remaining 16.7% (11/66) receiving the dose divided into two administrations per day.

A median loading dose of 93.7 mg/kg (range 51.3–551.2 mg/kg; IQR 64.6–126.7 mg/kg) was prescribed in 28.3% (26/92) of cases, split into two administrations per day in 61.5% of dogs. A single dose per day was prescribed in 26.9% (7/26) of cases, with the remaining 11.5% (3/26) receiving KBr every 8 h. Serum bromide concentrations were monitored in 31.6% of dogs. Detailed information on KBr dose can be found in Table 3.

Data completeness was high across the dataset. Age, sex, neuter status, weight, and breed were recorded for almost all dogs (>99.9%). The dose of each ASD was available for all dogs on ZNS prescriptions and for 98.7, 98.9 and 96.8% of those receiving PB, LEV and KBr, respectively. The frequency of administration of each ASD was available for 100% of dogs on ZNS and for 99.0, 98.1, and 96.8% of those receiving PB, LEV, and KBr, respectively (Tables 2, 3).

## Discussion

This retrospective study on canine IE provides valuable insights into the epidemiology and treatment choices for this chronic neurological disorder in primary care practices across the US. Remarkably, the study highlights a relatively low prevalence of IE in dogs between 6 months and 6 years of age, identifies the most common breeds diagnosed with IE, and assesses how closely treatment practices align with the 2015 ACVIM Small Animal Consensus Statement on Seizure Management in Dogs (12).

The estimated prevalence of IE in this study was 0.03%, which is significantly lower than the previously reported 0.6% in the canine population in the United Kingdom (1). The UK-based study investigated the prevalence of epilepsy of unknown origin (EUO) among dogs attending first-opinion practices. The diagnosis of EUO was based on a history of more than two seizures in the absence of other medical problems and a seizure history extending beyond 1 year, or alternatively, on documentation of four or more repeat ASD prescriptions (1). In contrast, the present study used narrower inclusion criteria to define IE, i.e., dogs aged 6 months to 6 years at their first recorded epileptic seizure, with normal clinical and neurological examinations, unremarkable routine blood tests, and documented ASD prescription. These inclusion criteria likely resulted in a smaller case population, contributing to the lower prevalence estimate observed in this US primary-care cohort.

A higher prevalence of 0.75% was reported in a large Swedish insurance claim–based cohort study; however, the Swedish population included dogs with epilepsy of any etiology, indicating that the true prevalence of IE was likely lower (27). Moreover, insurance datasets tend to reflect a subset of the canine population with higher healthcare utilization, potentially resulting in more frequent diagnostic work-up and case recording. These methodological differences contribute to the disparity in prevalence estimates between studies.

Several factors within the present study may have further contributed to underestimation of prevalence. The retrospective design and reliance on structured data extracted from electronic medical records meant that dogs with incomplete or unclear history of epileptic seizures were excluded. The inclusion criteria were aimed at avoiding the possibility of wrongly including patients affected by other conditions causing seizures or epileptic seizure-like episodes but not epileptic seizures (8, 28). As such, it is possible that dogs affected with IE might have been excluded due to incomplete documentation of the events in historical data. Furthermore, employing ASD prescription as an inclusion criterion may have resulted in the exclusion of dogs with IE who were not prescribed ASDs, either because of infrequent epileptic seizures, owner preference or prescription being issued outside Banfield practices. Additionally, to estimate population prevalence, the denominator consisted of all unique canine patients aged 6 months to 6 years presented during the study period. This age restriction was applied because it aligns with the typical onset window for IE (12). However, excluding dogs younger than 6 months and older than 6 years may have led to prevalence underestimation by omitting dogs whose epileptic seizure onset falls outside this expected range (12). Finally, the reported prevalence was derived from a single, albeit large, corporate network of veterinary clinics, and estimates may differ in populations that include both corporate and independently owned practices.

The breed distribution observed in this study partially aligns with the known breed predispositions in the veterinary literature (1, 27). However, since no comparison was made with the non-affected population, these findings represent breed frequencies within the IE cases rather than evidence of breed predisposition.

Labrador Retrievers were the most frequently affected breed in our cohort. These results are consistent with earlier reports, including a study on Danish Labrador Retrievers that identified a significant prevalence of IE in the breed (29, 30). Siberian Huskies and Chihuahuas were also largely represented. However, the current literature does not strongly support a breed-specific predisposition of these dogs to IE. Therefore, their presence in our study may be influenced by their popularity and high numbers in the general canine population in the US (31). Crossbreeds accounted for 6.6% of dogs in this study. Although mixed-breed dogs are rarely the focus of breed-specific epilepsy research, their inclusion provides important context. Most studies emphasize purebred populations due to their genetic uniformity and the possibility of tracing inheritance patterns within specific breeds. The presence of IE among crossbreeds in our cohort suggests that IE can frequently occur independently of breed predispositions, reflecting the diversity of the primary care canine population and offering a useful comparison group to evaluate the influence of genetic versus environmental factors in the development of epilepsy (32). Other overrepresented breeds in this cohort included Poodles, German Shepherds and Australian Shepherds, which is consistent with previous studies (1, 29).

Focusing on the treatment, PB was the most prescribed first-line ASD (34.9% of dogs included in this study). This finding is consistent with reports from Europe, the US, and Australia, where PB has also been identified as the most frequently prescribed ASD (33–35).

In this study, PB was typically prescribed every 12 h, with doses aligning with the ACVIM Consensus Statement recommendations, which advises a starting dose of approximately 2.5 mg/kg PO every 12 h (12). Therapeutic drug monitoring was performed in 77.5% of cases, demonstrating primary care veterinarians’ awareness of the need to monitor PB serum concentration to optimize treatment outcomes (i.e., maximize seizure control and minimize the risk of adverse events/toxicity).

Levetiracetam was the second most frequently prescribed ASD, prescribed to 31.3% of dogs, predominantly in the XR formulation. The wide dosing range observed for the XR preparation (most commonly administered every 12 h at 11.0–79.3 mg/kg) suggests variability in prescribing practices in primary care, likely reflecting differences in clinician preference. Extended-release levetiracetam was prescribed every 12 h in nearly 90% of dogs, which is consistent with pharmacokinetic recommendations (36). Notably, the 2015 ACVIM Consensus Statement does not provide a recommended starting dose for the XR formulation, which may further contribute to the heterogeneity observed in prescribing practices. Since the extended formulation is only available in a minimum tablet strength of 500 mg, accurate dosing in small-breed dogs can be challenging. Notably, Chihuahuas accounted for nearly 7% of the study population, making it plausible that some very small dogs received relatively high doses or required tablet splitting to approximate the intended dose. Splitting and crushing XR tablets compromises the integrity of the controlled-release matrix, which can affect the XR characteristics of the formulation (37, 38). This limitation should be considered when interpreting dosing practices in small dogs and may partially explain the variability in clinical response.

Zonisamide and KBr were prescribed less frequently, at 22.9 and 11.1% of the study population, respectively. Zonisamide dose and frequency of administration closely reflected the Consensus Statement guidelines (5 mg/kg every 12 h), with a median of 5.8 mg/kg every 12 h (12). Potassium bromide was typically prescribed once daily in maintenance therapy, with a median dose of 29.9 mg/kg/day. This value falls within the recommended range provided in the 2015 ACVIM Consensus Statement, which suggests a maintenance dose of 40 mg/kg/day for monotherapy in dogs (12). However, some variability was observed in this study, including higher doses and twice-daily administration. The ACVIM Consensus Statement does not provide a loading protocol for KBr and PB, with different doses being described in the literature (11, 39). In this study, a highly variable KBr loading dose (median: 93.7 mg/kg/day; IQR 64.6–126.7 mg/kg) was prescribed in 28.3% of cases, highlighting the absence of standardized guidelines for loading protocols. Finally, therapeutic drug monitoring for KBr was performed in only 31.6% of cases, suggesting an opportunity for improvement in monitoring practices.

In summary, these findings support that prescribing practices in Banfield primary care practices largely align with the ACVIM guidelines, particularly for ZNM and maintenance doses of PB and KBr (12). Levetiracetam and KBr loading doses showed substantial variability, underscoring the need for clearer, evidence-based dosing guidance. The apparent lack of therapeutic monitoring in 22.5% of dogs prescribed PB and in 68.4% of dogs receiving KBr may be attributable to the dog-owner’s preferences and financial constraints rather than the veterinarians’ recommendation. In addition, some owners may have pursued testing through another venue, indicating that the proportions observed in this dataset likely represent the minimum percentage of dogs undergoing therapeutic monitoring. Nevertheless, more education on the benefits of therapeutic drug monitoring may support veterinary and pet-owner’s decision making.

Furthermore, although the doses prescribed were consistent with ACVIM 2015 Consensus recommendations (12), clinical efficacy could not be evaluated. The lack of follow-up information limited the ability to determine whether the chosen doses were effective in individual dogs. As a result, dose appropriateness in this study reflects adherence to guidelines rather than demonstrated effectiveness of the ASD.

It is also worth noting that, although LEV and ZNM do not hold Food and Drug Administration approval for the treatment of IE in dogs, they were commonly prescribed extra-label in this population. Their widespread use in primary care practices might reflect clinical preferences based on perceived safety, ease of administration, and tolerability, rather than licensing status. Extended-release levetiracetam, for example, might be preferred over other ASDs due to the limited side effects, twice-daily administration and lack of hepatic metabolism, which may be advantageous in patients with comorbidities or when used alongside other medications (12). Similarly, ZNM is commonly prescribed due to its twice-daily dosing and low incidence of adverse effects (40). Recent research indicates that ZNM monotherapy is effective in managing newly diagnosed IE in dogs, with a substantial proportion of patients achieving seizure freedom or a ≥ 50% reduction in epileptic seizure frequency (40).

These prescribing patterns highlight the importance of updating veterinary licensing and treatment guidelines to reflect current clinical practices and new evidence.

This study has some limitations that should be taken into account. Case identification relied on structured electronic medical record fields, and free-text notes were not accessible, which restricted the inclusion of dogs with incomplete or unclear seizure histories. The requirement for an ASD prescription may also have narrowed the cohort by excluding dogs with IE whose epileptic seizures were infrequent or managed outside the Banfield network. Although this was outside the scope of the study, the lack of follow-up information prevented assessment of seizure characteristics, treatment response or adverse effects. Therapeutic drug monitoring performed outside Banfield practices was not captured, so the reported proportions represent minimum values. In addition, because the breed distribution of cases could not be compared with that of non-affected dogs, the study describes breed frequencies rather than breed predisposition. Finally, accurate dosing of XR levetiracetam was occasionally limited by tablet size constraints in very small dogs, meaning that some dose variability likely reflected practical formulation limitations rather than differences in prescribing intent.

In conclusion, certain breeds reported in the literature as being predisposed to epilepsy (1, 27) were also frequently represented among the IE cases in this study; however, this pattern may reflect underlying population demographics rather than a true breed predisposition. In most cases, the veterinary recommendation and care provided appeared to follow ACVIM Consensus Statement recommendations (12), suggesting good implementation of these guidelines. Nonetheless, the variability in LEV and KBr dosing, along with the frequent extra-label use of ASDs in the US, highlights the need for updated evidence-based dosing recommendations and for revisions to veterinary licensing and treatment guidelines that incorporate current clinical practice and new scientific evidence. Finally, increasing awareness of the value of therapeutic drug monitoring could help guide both veterinary recommendations and owner choices in managing canine IE.

## Data Availability

The raw data supporting the conclusions of this article will be made available by the authors, without undue reservation.
